# Cost-effectiveness analysis of health tapestry, a complex primary care program for older adults: a post-hoc analysis

**DOI:** 10.1186/s12875-024-02475-5

**Published:** 2024-07-03

**Authors:** J. E. Tarride, G. Blackhouse, L. Lamarche, P. Forsyth, D. Oliver, T. Carr, M. Howard, L. Thabane, J. Datta, L. Dolovich, R. Clark, D. Price, D. Mangin

**Affiliations:** 1 Department of Health Research Methods, Evidence and Impact, McMaster University, Hamilton, ON Canada; 2 Programs for Assessment of Technologies in Health, St Joseph’s Healthcare Hamilton, Hamilton, ON Canada; 3 Department of Family Medicine, McMaster University, Hamilton, ON Canada; 4 School of Kinesiology and Health Science, York University, Toronto, Canada; 5 Biostatistics Unit, St Joseph’s Healthcare Hamilton, Hamilton, ON Canada; 6 Leslie Dan Faculty of Pharmacy, University of Toronto, Toronto, ON Canada

**Keywords:** Cost-Effectiveness, Health TAPESTRY, Volunteers, Family Health Team, Elder Care

## Abstract

**Background:**

We initially reported on the cost-effectiveness of a 6-month randomized controlled implementation trial which evaluated Health TAPESTRY, a primary care program for older adults, at the McMaster Family Health Team (FHT) site and 5 other FHT sites in Ontario, Canada. While there were no statistically significant between-group differences in outcomes at month 6 post randomization, positive outcomes were observed at the McMaster FHT site, which recruited 40% (204/512) of the participants. The objective of this post-hoc study was to determine the cost-effectiveness of Health TAPESTRY based on data from the McMaster FHT site.

**Methods:**

Costs included the cost to implement Health TAPESTRY at McMaster as well as healthcare resource consumed, which were costed using publicly available sources. Health-related-quality-of-life was evaluated with the EQ-5L-5L at baseline and at month 6 post randomization. Quality-adjusted-life-years (QALYs) were calculated under an-area-under the curve approach. Unadjusted and adjusted regression analyses (two independent regression analyses on costs and QALYs, seemingly unrelated regression [SUR], net benefit regression) as well as difference-in-difference and propensity score matching (PSM) methods, were used to deal with the non-randomized nature of the trial. Sampling uncertainty inherent to the trial data was estimated using non-parametric bootstrapping. The return on investment (ROI) associated with Health TAPESTRY was calculated. All costs were reported in 2021 Canadian dollars.

**Results:**

With an intervention cost of $293/patient, Health TAPESTRY was the preferred strategy in the unadjusted and adjusted analyses. The results of our bootstrap analyses indicated that Health TAPESTRY was cost-effective compared to usual care at commonly accepted WTP thresholds. For example, if decision makers were willing to pay $50,000 per QALY gained, the probability of Health TAPESTRY to be cost effective compared to usual care varied from 0.72 (unadjusted analysis) to 0.96 (SUR) when using a WTP of $50,000/QALY gained. The DID and ROI analyses indicated that Health Tapestry generated a positive ROI.

**Conclusion:**

Health TAPESTRY was the preferred strategy when implemented at the McMaster FHT. We caution care in interpreting the results because of the post-hoc nature of the analyses and limited sample size based on one site.

## Background

As the proportion of older adults increase, and in parallel multimorbidity, there is a need to strengthen primary care systems through primary care programs to improve patient outcomes among older adults while optimizing resource allocation. Such a program is Health TAPESTRY (Health Teams Advancing Patient Experience: STRengthening qualitY) which was initially piloted in a randomized controlled trial (RCT) conducted at the McMaster Family Health Team (FHT) site in Hamilton, Ontario, Canada [1]. Results showed that patients who received Health TAPESTRY spent significantly less time sitting, walked more, had fewer hospitalizations, and saw their primary health care team more often at 6-month follow-up compared to usual care in the absence of the Program [1]. Based on these promising results, a second RCT studying the effectiveness of broader implementation using the RE-AIM framework [2] was conducted from March 2018 to August 2019 to determine whether the effectiveness of Health TAPESTRY seen in the original trial conducted in an academic site could be replicated in other health care settings. The study protocol [3] and main clinical and economic results have been described elsewhere.

This pragmatic 1:1 parallel group randomized control trial (RCT) was conducted at the McMaster FHT site and five other FHT sites in Ontario. All participants were at least 70 years old and were rostered to a participating physician. Briefly, Health TAPESTRY consists of four main parts: 1) trained volunteers to connect with clients in their home to gather health and social information from clients (i.e., patients enrolled in Health TAPESTRY); 2) interprofessional primary health care teams providing health care services to clients and working with them on meeting their health goals; 3) technology to collect and share information; and 4) community engagement and connections to create links between clients and resources in their communities [3]. While results showed that Health TAPESTRY was successfully implemented in the six sites, the benefits of the intervention on hospitalizations and physical activity observed in the original trial were not replicated [4]. With intervention costs of $562 per patient, a trial-based economic evaluation of Health TAPESTRY indicated that the incremental cost-effectiveness of Health TAPESTRY compared to usual care was approximately $150,000 per quality adjusted life years (QALYs) gained [4].

However, post-hoc analyses [4] indicated that when compared to usual care, Health TAPESTRY had a beneficial impact on hospitalizations, emergency room visits and health-related quality of life (HRQoL) at the McMaster FHT, the main trial site with approximately 40% of the trial participants. Several factors may explain these differences. First, the McMaster FHT was the site who conducted the pilot study evaluating Health TAPESTRY and, there may have been learnings from the pilot trial that may have positively affected outcomes [4]. Second, there were differences in healthcare provider teams or clinic workflows between the McMaster FHT site and other FHT sites. For example, the McMaster site was the only site with a physiotherapist and a system navigator and one of only two sites with multiple registered practical nurses, which may have impacted healthcare planning, delivery and outcomes. From an economic point of view, since the McMaster FHT site recruited 40% of the trial population, it should be expected that the intervention cost per patient would be lower at the McMaster site than in the other sites which recruited less patients. To provide additional insights into the costs and benefits of Health TAPESTRY when implemented in an “ideal” setting where a large number of patients were recruited, a post-hoc economic evaluation of Health TAPESTRY was conducted using data from the McMaster FHT site.

## Methods

### Study design and participants

A trial-based economic evaluation was conducted to determine the cost-effectiveness of Health TAPESTRY when implemented at the McMaster FHT site. The study design and results of the Health TAPESTRY trial and the broader aspects of implementation are described in detail elsewhere. Briefly, the Health TAPESTRY was a pragmatic 1:1 RCT which randomized 512 people (257 Health TAPESTRY and 255 controls), across 6 FHT sites of which the majority were recruited from the McMaster FHT site (*n* = 100 in the Health TAPESTRY arm and *n* = 104 in the control arm). Patient-reported health outcomes and healthcare resource utilization were collected in the trial and analyzed at 6 months post randomization [3, 4].

### Intervention costs, healthcare resource utilization and unit costs

As discussed elsewhere [3, 4], Health TAPESTRY intervention costs were estimated using administrative financial information from the trial and included costs related to personnel, office supplies and equipment, tablets, travel, volunteer onboarding, Tap-App (i.e. Health TAPESTRY App to collect data) maintenance costs, and Vimeo (i.e., system supporting videos embedded in the online volunteer training) licencing costs.

Due to the design of the trial [3] in which the usual care group received the Health TAPESTRY intervention at 6 months, the Health TAPESTRY intervention costs included the costs associated with the group who were randomized to the intervention at baseline and those who received the intervention at 6 months. In addition, there were several couples who received the intervention while only one participant per household was randomized [4]. As such, Health TAPESTRY intervention costs per patient were derived by dividing the overall trial intervention costs by the total number of participants who received the intervention (e.g., randomized population and spouses/partners). Intervention costs considered in this economic evaluation were those related specifically to the McMaster FHT site.

Six-month health care resource utilization before the baseline visit, and between the baseline visit and the study visit at 6 months were derived from patient electronic medical records. Health care utilization included in the economic evaluation included the number of hospitalizations, number of emergency room visits, and the number of primary care visits. Although medication use was also collected as part of the trial, the economic evaluation did not consider medication costs as the information on the dosing of the medications and treatment duration were not captured as part of the trial. Health care utilization costs were calculated for each patient by applying unit costs to their respective utilization data. As described elsewhere [4], the cost per hospitalization ($6,610) [5] and ER visit ($319) [6] were based on data from the Canadian Institute for Health Information. The cost per primary care visit ($38) was based on the cost of a general practitioner re-assessment from the Ontario Schedule of Physician Benefits [7]. All costs are expressed in 2021 Canadian dollars unless otherwise specified.

### Health-related quality of life and quality-adjusted-life-years

The EQ-5D-5L instrument was administered in the trial during the baseline and 6-month visit to measure HRQOL. The Canadian scoring algorithm [8] was used to generate patient-level EQ-5D-5L utility scores at baseline and at 6 months. Quality-adjusted-life-years (QALYS) were calculated for each patient using an area under the curve approach. QALYs combine quantity of life and quality of life (QoL), where QoL is expressed in terms of utilities in a 0–1 scale (0 represents death and 1 perfect heath). When using a time horizon of 6 months, the maximum number of QALY gained is 0.5 (0.5 year of life with a utility of 1).

### Statistical and cost-effectiveness analyses

As per the main trial results [4], the study population enrolled at the McMaster FHT site was described in terms of age, sex, ethnicity, marital status, income, and Charlson comorbidity index (CCI) scores using mean and standard deviation (SD) or percentages. Differences in baseline characteristics between the Health TAPESTRY and usual care groups at the McMaster FHT site were assessed using chi-square tests for categorical variables and student t-tests for continuous variables.

As recommended by the economic guidelines from the Canadian Agency for Drugs and Technologies in Health (CADTH), a cost-utility analysis was conducted to compare the interventions in terms of costs and QALYs [9]. In addition to an unadjusted analysis comparing costs and QALYs between the Health TAPESTRY and usual care groups, several cost-effectiveness methods [10] were used to deal with the non-randomized nature of the data. The first approach was based on using two independent multivariable regressions to adjust 6-month costs and QALYs by baseline values (i.e., baseline cost for the cost regression and baseline utility for the QALY regression), age, sex, and Charlson Comorbidity Index (CCI) score [10]. When using this approach, a generalized linear model with log link and gamma distribution was used for modeling costs [11, 12] while an ordinary linear square (OLS) regression was used for the QALYs regression [13]. Adjusting for the same covariates, our second economic approach used seemingly unrelated regressions (SUR) to simultaneously solve the costs and QALYs regression equations [11]. In contrast to the former approach, which was based on independent regressions, SUR have the benefit to account for correlation between costs and QALYs when adjusting for baseline characteristics. Our third approach was to use propensity score matching (PSM) methods [14, 15] to create comparable cohorts of individual’s receiving Health Tapestry and usual care. Variables included in the score included age, gender, ethnicity, marital status, income level and Charlson Comorbidity Index score. A greedy nearest-neighbor 1:1 matching algorithm was used with a caliper of 0.10 width of the standard deviation (SD) of the logit of the propensity score. Balance in baseline covariates for each cohort was evaluated using standardized differences [16]. Our last economic approach to analyze this non-randomized data is based on the net benefit (NB) regression framework [17] when adjusting for baseline characteristics. As opposed to the previous approaches which determine the cost-effectiveness of Health TAPESTRY compared to usual care in terms of incremental cost per QALY gained, the outcome of the NB regression method is expressed in terms of incremental net monetary benefit. If the net monetary benefit is positive at a certain willingness to pay per one unit of QALY gained, then the intervention is considered cost-effective. Two commonly cited willingness to pay values of $50,000 and $100,000 per QALY gained were used when estimating the incremental monetary net benefit of Health TAPESTRY. As described in the study protocol [3] and main trial results [4], multiple imputations [12] were used to impute missing data. Results are presented in terms of means, standard errors (SEs) and 95% confidence intervals (CIs) for the costs and QALYs differences based on 1,000 bootstrap simulations.

For each approach (unadjusted analyses, two-independent multivariable regressions, multivariable SUR, and net benefit regression adjusting for baseline values), sampling uncertainty was estimated using non-parametric bootstrapping [12] and the uncertainty was presented using cost-effectiveness acceptability curves (CEACs) [18]. CEACs show the probability that an intervention is cost-effective for various willingness to pay thresholds. Results are presented in terms of means, standard errors (SEs) and 95% CIs for the costs and QALYs differences based on 1,000 bootstrap simulations.

Additionally, a difference-in-difference approach (DID) [19–21] was used where the 6-month pre-baseline and the 6-month post-baseline healthcare resource utilization costs (i.e. ED, hospitalization, and primary care visits) were first calculated for each intervention. The resulting DID estimate was then bootstrapped to generate a 95% CIs around the DID cost estimate. Since there was no information on the QALYs prior to the intervention, only costs were analyzed under the DID approach. In addition to the cost-effectiveness analyses, a cost–benefit analysis was conducted to determine the return on investment (ROI) associated with the intervention. The ROI was determined as the difference in healthcare cost savings associated with Health TAPESTRY and the Health TAPESTRY intervention cost divided by the Health TAPESTRY intervention costs.

To test the robustness of the results against changes in key assumptions, several one-way sensitivity analyses were conducted: 1) Intervention costs based on the number of randomized patients only (as opposed to include both randomized patients and their partners who received the intervention); 2) unit costs decreased by 20%; and 3) unit costs increased by 20%. The analyses were conducted and reported according to Canadian and international guidelines for economic evaluations [9, 12, 22].

## Results

### Baseline demographics

Table 1 presents the baseline characteristics of the participants enrolled at the McMaster FHT site in the Health TAPESTRY (*n* = 100) and control (*n* = 104) groups. Despite the non-randomized nature of the data, there were no statistically significant differences between the Health TAPESTRY and usual care groups in terms of age, female participants, marital status, income levels, or CCI score.

**Table 1:** Baseline characteristics of participants enrolled in the McMaster FHT site

	**Health TAPESTRY** (***n*** **= 100)**	**Usual Care (*n*** **= 104)**	***p*-value**
Age, year mean (SD)	77.1 (5.5)	76.8 (5.7)	0.67
Female, n (%)	65 (67.7%)	64 (66.0%)	0.61
European or white ethnicity, n (%)	84 (87.5%)	83 (85.6%)	0.59
Marital Status, n (%)			0.98
Married or common Law	41 (42.7%)	38 (39.2%)	
Divorce, separated widowed, never married	55 (57.3%)	59 (60.8%)	
Household income n (%)			0.67
Under $20,000	6 (7.4%)	11 (13.1%)	
$20,001 to $50,000	34 (42.0%)	32 (38.1%)	
$50,001 to $70,000	15 (18.5%)	20 (23.8%)	
$70,001 to $100,000	14 (17.3%)	13 (15.5%)	
$100,001 to $150,000	10 (12.4%)	6 (7.1%)	
Greater than $150,000	2 (2.5%)	2 (2.4%)	
Charlson Comorbidity Index, mean (SD)	1.01 (1.2)	1.30 (1.6)	0.22

Notation: *SD* standard deviation

### Healthcare resource utilization and utility

Table 2 presents the healthcare resource utilization and EQ-5D-5L utility associated with the Health TAPESTRY and usual care groups at the McMaster FHT site. Although there were differences in the baseline values between the intervention and the usual care groups, the number of hospitalizations and ED visits decreased in the intervention group between baseline and month 6. On the other hand, the number of primary care visits increased in the intervention group. In terms of HRQoL, the utility scores slightly improved in both the intervention and usual care.

**Table 2:** Healthcare resource utilization and utility at baseline and Month 6 (McMaster FHT site)

	**Health TAPESTRY (*n*** **= 100)**		**Usual Care (*n*** **= 104)**	
	Baseline	Month 6	Baseline	Month 6
Hospitalizations: mean (SE)	0.23 (0.06)	0.10 (0.04)	0.14 (0.04)	0.16 (0.04)
Emergency department or urgent care visits: mean (SE)	0.33 (0.11)	0.20(0.09)	0.23 (0.06)	0.27 (0.08)
Primary care visits: mean (SE)	2.71 (0.36)	3.65 (0.39)	3.31 (0.30)	3.40 (0.46)
EQ-5D-5L utility: mean (SE)	0.80 (0.02)	0.82 (0.02)	0.80 (0.02)	0.81 (0.02)

Notation: *SE* standard error

### Cost-effectiveness and cost–benefit analyses

The intervention cost per participant at the McMaster FHT site was $293 (Table 3). The results of the unadjusted analyses indicated that the 6-month total mean (SE) costs were $1,185 ($255) for Health TAPESTRY and $1,299 ($308) for usual care, resulting in a difference of $114 (95% CI: -$428; $246). Most of these savings were due to a decrease in hospitalizations seen in the Health TAPESTRY group. As a consequence, the ROI was positive at 0.39 (i.e., 39% return on investment). The mean (SEs) number of QALYs over the 6-month period were 0.404 (0.007) for the intervention and 0.403 (0.008) for the usual care group, for a difference of 0.001 (95% CI: -0.0074; 0.0094) QALYs in favor of the intervention. Table 4 presents these results. Similar results (i.e., Health TAPESTRY less costly and more effective than usual care) were observed in our regressions when costs and QALY’s were adjusted for baseline values, age, sex and CCI score or when the PSM method was used (Table 5). The Appendix presents the characteristics of our matched cohorts using the PSM (N = 75 each). The results of our DID approach also confirmed that Health TAPESTRY was cost saving as indicated by DID cost estimates of $742 savings for Health TAPESTRY (95% CI: $292, $1,254) when using the full trial data and $325 (95% CI: -$215, $840) when using the sample resulting from the PSM. The resulting ROI based on the DID method were 2.5 and 1.1.

**Table 3:** Health TAPESTRY intervention unit costs in 2021 Canadian dollars

Cost Item	McMaster FHT Costs
Personnel	$27,273
Office Supplies and equipment	$7,247
Tablets	$11,025
Travel	$2,550
Volunteer Onboarding	$18,635
TAP-App	$2104
Vimeo Licencing costs	$639
Total	$69,473
Number of patients^a^	237
Cost per patient	$293.13

^a^Includes both intervention and control patients as control patients were given intervention after 6 months. Includes 23 individuals from couples who were enrolled at the McMaster FHT site but were not randomized

**Table 4:** Cost-effectiveness results in 2021 Canadian dollars: unadjusted analyses

	**Health TAPESTRY:** **Mean (SE)**	**Usual Care:** **Mean (SE)**	**Difference** **Mean (95%CI)**
Health TAPESTRY intervention costs	$293	$0	$293
Hospitalization costs	$688 ($253)	$1082 ($296)	-$394 (-$715; -$67)
Emergency department costs	$64 (27)	$87 ($24)	-$23 (-$45; $1)
Primary care visit costs	$140 ($15)	$130 ($17	$10 (-$5; $23)
Total Costs	$1,185 ($255)	$1,299 ($308)	-$114 (-$428; $246)
Quality adjusted life years (QALYs)	0.4040 (0.007)	0.4028 (0.008)	0.0012 (-0.0074; 0.0094)
Return on investment [ (healthcare costs savings– intervention costs) / intervention costs]			0.39

Notations: *SE* Standard error; *95% CI* 95% confidence interval

**Table 5:** Cost-effectiveness results in 2021 dollars (Health TAPESTRY versus usual care)

	**Incremental Costs** **Mean (95% CIs)**	**Incremental QALYs** **Mean (95% CIs)**	**ROI**	**Probability that Health TAPESTRY is cost-effective**^**a**^ **when the willingness to pay is:**	
				**$50,000/QALY**	**$100,000/QALY**
Unadjusted analysis	-$114 (-$428; $246)	0.0012 (-0.0074; 0.0094)	0.39	0.72	0.68
Two independent regressions	-$100 (-$417; $324)	0.0034 (-0.0005; 0.0074)	0.34	0.84	0.97
Seemingly unrelated regressions	-$187 (-$502; $133)	0.0034 (-0.0005; 0.0073)	0.64	0.96	0.90
PSM method	-$52 (-$435; $347)	0.0074 (-0.0023; 0.0164)	0.18	0.88	0.92
Net benefit regression^b^				0.92	0.90

Notations: *95% CI* 95% confidence interval, *NMB* net monetary benefit, *QALY* quality-adjusted-life-years, *PSM* propensity scoring matching, *ROI* return on investment

^a^Based on 1,000 bootstrap to deal with the sampling uncertainty associated with the trial

^b^The net monetary benefit is positive when willingness to pay per QALY is $50,000 (NMB = $280) and $100,000 (NMB = $384) showing that Health TAPESTRY is cost-effective based on point estimates

**Fig. 1:**
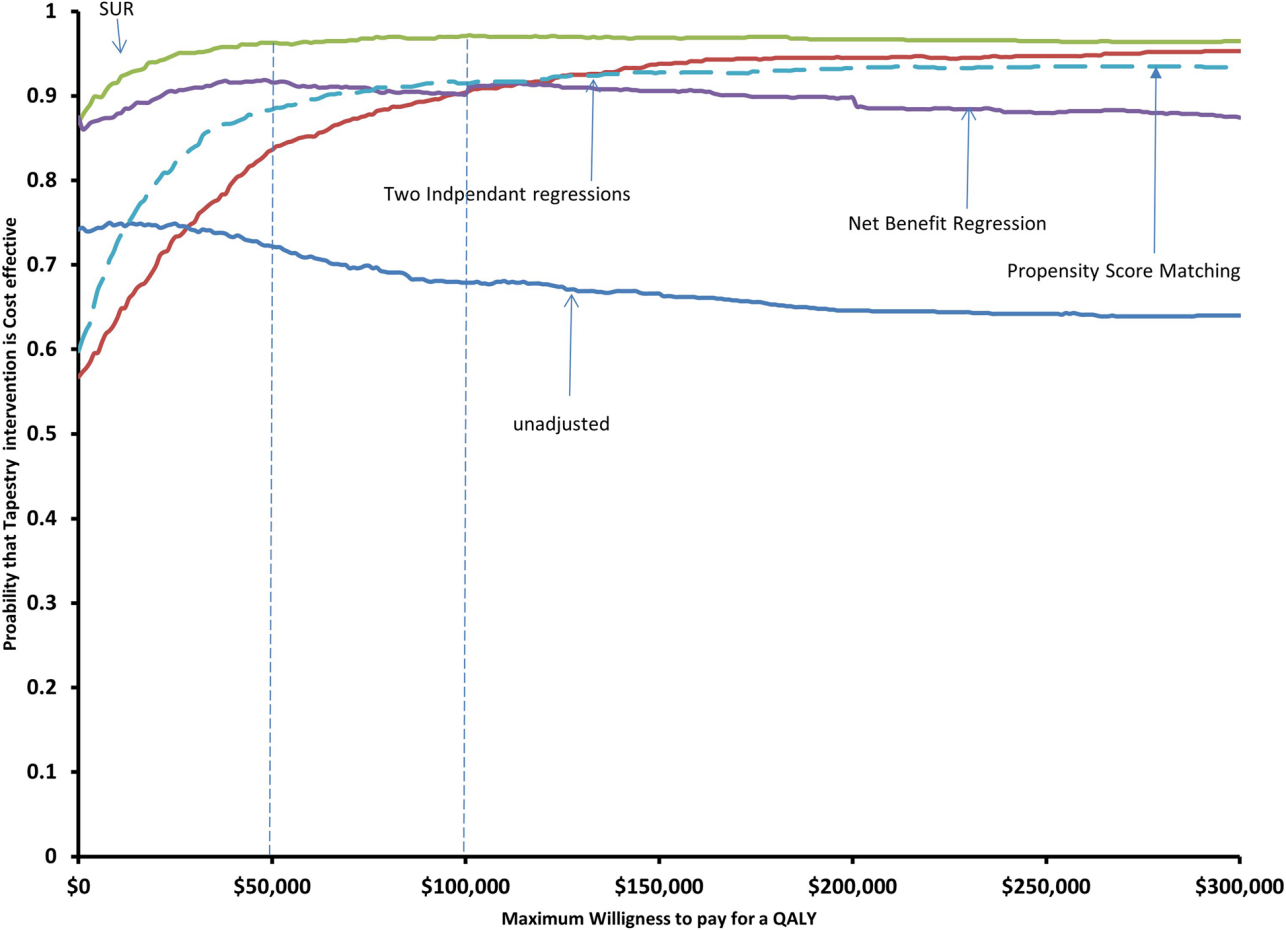
Cost effectiveness acceptability curves (CEAC’s)

While the point estimates seem to indicate that Health TAPESTRY was less costly and produced more QALYs than usual care, there was uncertainty around the incremental costs and QALYs estimates as reflected in the 95% CIs (Table 4). However, the results of the bootstrapped analyses conducted to deal with the sampling uncertainty inherent to the trial data indicated that Health TAPESTRY was cost effective at commonly cited willingness to pay thresholds. As shown in Table 5, the probability of Health TAPESTRY to be cost effective compared to usual care varied from 0.72 (unadjusted analysis) to 0.96 (SUR) when using a WTP of $50,000/QALY gained. These probabilities range from 0.68 (unadjusted analysis) to 0.97 (SUR) if decision makers were willing to pay $100,000 per QALY gained. Figure 1 presents the CEACs associated with each of our four methods to determine the cost-effectiveness of Health TAPESTRY (unadjusted analyses, two independent multivariable regressions, seemingly unrelated multivariable regressions, multivariable net benefit regression). The results of our one-way sensitivity analyses presented in Table 6 shows that changing key assumptions did not change the conclusions. Health TAPESTRY was the dominant strategy in all scenarios, except when using the PSM method and assuming health care costs were 20% lower than the base case. Even in this case, with an incremental cost-effectiveness of less than $3,000/QALY gained, Health TAPESTRY was the preferred strategy.

**Table 6:** Sensitivity Analyses (Health TAPESTRY versus control/no program)

Sensitivity Analyses	Incremental Costs	Incremental QALYS	Incremental Cost-Effectiveness Statistic	ROI
Intervention cost = $340.55				
Unadjusted	-$66	0.0012	Dominates^a^	0.19
Seemingly unrelated regressions	-$148	0.0034	Dominates^a^	0.43
Two Independent regressions	-$45	0.0034	Dominates^a^	0.13
PSM	-$4	0.0074	Dominates^a^	0.01
Unit Costs 20% higher				
Unadjusted	-$195	0.0012	Dominates^a^	0.67
Seemingly unrelated regressions	-$274	0.0034	Dominates^a^	0.94
Two Independent regressions	-$189	0.0034	Dominates^a^	0.65
PSM	-$120	0.0074	Dominates^a^	0.41
Unit Costs 20% lower				
Unadjusted	-$32	0.0012	Dominates^a^	0.11
Seemingly unrelated regressions	-$101	0.0034	Dominates^a^	0.34
Two Independent regressions	-$12	0.0034	Dominates^a^	0.04
PSM	$17	0.0074	$2,298/QALY	-0.06

Notations: *PSM* propensity scoring matching, *ROI* return on investment

^a^Health TAPESTRY dominates usual care as it is less costly and more effective

## Discussion

Consistent with the post-hoc analysis [4], the results of this economic evaluation using data from the McMaster FHT site showed that Health TAPESTRY was the preferred strategy compared to usual care. The results were consistent between the five different methods used to analyse the data (i.e. unadjusted, two independent regressions, sur, DID and PSM). This contrasts with the main trial-economic evaluation which found an incremental cost-effectiveness ratio of approximately $150,000 per QALY gained when the data from McMaster FHT site and the other five FHT sites were analyzed together [4]. Compared to the five other FHT sites, positive outcomes and lower intervention costs were associated with Health TAPESTRY at the McMaster FHT site [4]. Differences in healthcare provider teams of clinical workflow between the McMaster FHT site and the five other FHT sites may explain these differences. Finally, due to the relatively large number of patients recruited at the McMaster site, the intervention cost per patient to deliver Health TAPESTRY at the McMaster FHT site ($293 per patient) was approximately 50% lower that the intervention costs derived when using all trial data ($562 per patient) [4], which improved cost-effectiveness. For example, if the intervention cost per patient observed at McMaster was applied to the full trial data [4], then the cost-effectiveness of Health TAPESTRY compared to usual care would have been $45,000/QALY gained instead of $150,000/QALY gained.

It is difficult to compare our results with other economic evaluations of similar interventions as, to the best of our knowledge, there are no comparable primary care interventions which have integrated the different components of Health TAPESTRY. Nonetheless, an economic evaluation of a cluster RCT comparing a patient-centered approach to managing patients with multiple chronic conditions in England and Scotland (3D approach: 6-month general practitioner consultations, medication review and nurse appointments) compared to usual care reported an incremental cost-effectiveness ratio of £18,500 per QALY gained [23]. However, as noted by the authors, the results were subject to uncertainty and characterised by small incremental costs (£126) and QALYs (0.007) associated with the 3D approach [23], which is similar to our results (e.g. incremental QALYs (0.003). Due to different costing methods and healthcare systems, it is difficult to compare our cost results with this study. However, our uncertainty analyses showed that the probability of Health TAPESTRY to be cost-effective at a willingness threshold of $50,000/QALY gained varied from 0.7 to 0.9. As such, despite the uncertainty around the incremental costs and QALYs (e.g., 95% CIs include 0), Health TAPESTRY was the preferred strategy compared to usual care in all scenarios examined with our different methods.

Even though this is a post-hoc analysis, our economic evaluation of Health TAPESTRY using data from McMaster FHT site has several strengths. First, the McMaster site recruited 40% of the overall trial population (*N* = 204 out of 512), which allowed us to conduct an analysis at that site. Second, despite the non-randomized nature of the trial, the key baseline characteristics of the population enrolled at the McMaster FHT site were similar between the intervention and the usual care groups. Third, in addition to an unadjusted analysis, we used several techniques to recognize the non-randomized nature of the trial. While differences in numeral values of the results were seen between the different models due to different model assumptions (normal versus gamma distributions to model the costs), the results of this post hoc analysis showed that the Health TAPESTRY intervention was the preferred strategy compared to usual care in all analyses using the McMaster FHT site data. Although Health TAPESTRY was not generating cost savings when we changed the unit costs in the PSM method, Health TAPESTRY was highly cost-effective with an incremental cost of less than $3,000 per QALY gained compared to the status quo. Fourth, we conducted sensitivity analyses (e.g., changing unit costs by ± 20%) and the conclusions did not change. Despite these strengths, our study has several limitations. While we tried to adjust for age, sex and CCI score in our regression analyses or by using PSM methods, it is possible that there are unmeasured confounders that were not accounted for. While we used DID techniques, we could not verify the assumption related to the parallel trend assumption [11, 21] required for DID studies as we only had one data point before and after the intervention. Since we did not have access to detailed information on medications, we did not model the costs associated with medications. We conducted the analyses from a payer perspective and did not include any costs borne by patients or their caregivers (e.g., parking or productivity costs). We did not include the cost of volunteer time in our estimates. Replicating this intervention would require a volunteer organization to donate their services, otherwise additional costs would be incurred. Despite our best efforts to deal with the non-randomized nature of the analyses by using several statistical methods, it should be mentioned that this was a post hoc analysis and that the trial was not designed to analyze the McMaster FHT in isolation. Although there were no differences in key baseline characteristics between the groups at the McMaster FHT site and while the results were consistent across all statistical models, the risk for potential unmeasured confounding remains. While Health TAPESTRY was a complex intervention which included several components, it was not possible to determine the relative effect and cost-effectiveness of the various components of Health TAPESTRY. Finally, since this was a trial-based economic evaluation, we did not extrapolate the costs and benefits associated with the interventions beyond the trial duration of 6 months. This is left for future research.

Despite these limitations, the results of this post hoc analysis of the Health TAPESTRY intervention conducted at the McMaster FHT site showed that the positive outcomes seen at this academic site in both the initial and this trial also translated into savings and improvement in HRQoL despite the additional costs associated with the intervention. The full trial data economic analysis estimated the cost per QALY of the Health TAPESTRY Intervention to be $150,000 [4]. This paper adds to the economic analysis of the full trial as it is based on data from the primary family Health Team where the intervention may have been most optimally implemented. These results suggest that under certain conditions Health TAPESTRY could be both effective and cost effective.

Most importantly, from a policy point of view, the results highlight the need for careful understanding of how to successfully spread implementation of this complex intervention in primary care sites, rather than assuming RCT results in a single setting will translate across different settings. The results of this post-hoc analyses indicated that under “ideal” conditions, Health TAPESTRY saves costs and improve outcomes. The results also showed that the intervention cost per patient is a key driver of cost-effectiveness (i.e. when using the intervention costs per patient observed at McMaster FHT, Health TAPESTRY is cost-effective when using data from all 6 sites). Therefore, efforts should be made to minimize the intervention costs per patient when implementing programs such as Health TAPESTRY. However, it is expected that the cost of future implementations of Health TAPESTRY will be lower in a non-research environment which would improve cost-effectiveness.

## Conclusions

In this post-hoc analysis of the Health TAPESTRY trial at the McMaster FHT site, Health TAPESTRY was found to be both more effective and less costly than usual care. Strategies to support the wider implementation of Health TAPESTRY in the community are warranted.

## Data Availability

There are legal restrictions to sharing a de-identified data set. The reasons are outlined here: The original data is held by the respective hospitals, and data sharing agreements with the involved institutions that contributed data prohibit the authors from making the dataset publicly available. The full dataset creation plan is available from the authors upon request. For requests for de-identified and specified data access, contact the faculty in charge of the dataset at tarride@mcmaster.ca or mangind@mcmaster.ca.

## Appendix

**Table:** Baseline characteristics of the matched cohorts

	**Health TAPESTRY (*****n*** **= 75)**	**Usual Care** **(*****n *****= 75)**	**Standardized mean difference**
Age, year mean (SD)	77.2 (5.3)	77.3 (6.1)	0.02
Female, n (%)	49 (65.3%)	53 (70.7%)	0.11
European or white ethnicity, n (%)	64 (85.3%)	67 (89.9%)	0.11
Marital Status, n (%)			0.05
Married or common Law	31 (41.3%)	29 (38.7%)	
Divorce, separated widowed, never married	44 (58.7%)	46 (61.3%)	
Household income n (%)			
Under $20,000	4 (5.3%)	8 (10.7%)	0.18
$20,001 to $50,000	31 (41.3%)	29 (38.7%)	0.05
$50,001 to $70,000	17 (22.7%)	17 (22.7%)	0.00
$70,001 to $100,000	13 (17.3%)	11 (14.7%)	0.07
$100,001 to $150,000	7 (9.3%)	6 (8.0%)	0.05
Greater than $150,000	3 (4.0%)	4 (5.3%)	0.06
Charlson Comorbidity Index, mean (SD)	0.97 (1.1)	0.99 (1.4)	0.02

## Abbreviations

CEAC: Cost Effectiveness Acceptability Curve
CCI: Charlson Comorbidity Index
CI: Confidence Interval
DID: Difference-in-Difference
EQ-5D: EuroQOL-5D
FHT: Family Health Team
HRQOL: Health Related Quality of Life
PSM: Propensity Scoring Matching
QALYs: Quality Adjusted Life Years
RCT: Randomized Controlled Trial
SD: Standard Deviation
SE: Standard Error
SUR: Seemingly Unrelated Regressions
TAPESTRY: Health Teams
Advancing Patient Experience: STRengthening qualitY
